# Essential Update 2023/2024: Multidisciplinary Treatment for Invasive Intraductal Papillary Mucinous Carcinoma

**DOI:** 10.1002/ags3.70029

**Published:** 2025-04-21

**Authors:** Seiko Hirono

**Affiliations:** ^1^ Division of Hepato‐Biliary‐Pancreatic Surgery, Department of Gastroenterological Surgery Hyogo Medical University Nishinomiya Japan

**Keywords:** invasive IPMC, multidisciplinary treatment, neoadjuvant therapy, postoperative adjuvant therapy, recurrence

## Abstract

Invasive intraductal papillary mucinous carcinoma (IPMC) has a high malignant potential, with surgical resection being the only potentially curative treatment. However, even after surgical resection, recurrence occurs frequently and the prognosis is poor once recurrence develops. While retrospective studies aiming to achieve long‐term survival in invasive IPMC patients have been reported, the rarity of invasive IPMC has resulted in small‐scale datasets, leading to low levels of evidence. Consequently, the utility of adjuvant therapy after surgery, neoadjuvant therapy (NAT) before surgery, and treatments for postoperative recurrence in invasive IPMC remains unclear, with treatment strategies varying by institution—ranging from surgical resection alone to approaches based on conventional pancreatic cancer treatment. Recently, several large‐scale multicenter studies on invasive IPMC have been reported. These studies suggested that while adjuvant therapy after surgery may not be beneficial for all invasive IPMC patients, it could potentially extend survival in cases with advanced‐stage disease. Regarding NAT before surgery for invasive IPMC, the number of reported cases is extremely limited, and no definitive evidence has been established. For postoperative recurrence of invasive IPMC, some studies have indicated that administering treatment may prolong survival. Although these large‐scale studies have gradually clarified certain characteristics of invasive IPMC, they are all retrospective in nature, resulting in a low level of evidence. To achieve long‐term survival for invasive IPMC patients, large‐scale prospective multicenter studies are needed in the future.

## 1 Introduction

Intraductal papillary mucinous neoplasm (IPMN) is pathologically classified into low‐grade dysplasia (LGD), corresponding to adenoma; high‐grade dysplasia (HGD), corresponding to carcinoma in situ; and invasive intraductal papillary mucinous carcinoma (IPMC) [1]. Once it progresses to invasive IPMC, it can infiltrate the stromal lymphatic and blood vessels, as well as nerves, causing lymph node metastasis, distant metastasis, and nerve plexus invasion around major vessels. Surgical resection for invasive IPMC has been shown to improve survival, and currently, surgical resection is considered the only curative treatment for invasive IPMC [1]. However, even after surgical resection, recurrence is frequently observed [2, 3, 4, 5]. Although the incidence of invasive IPMC is lower compared to conventional pancreatic cancer, specific multimodal treatment strategies for invasive IPMC have yet to be established.

Adjuvant therapy after surgery for conventional pancreatic cancer has been proven to significantly prolong survival compared to surgery alone, as demonstrated by the CONKO‐001 randomized clinical trial (RCT) [6]. Subsequently, postoperative adjuvant therapy has become the standard treatment for conventional pancreatic cancer. However, the optimal regimen and duration of postoperative adjuvant therapy even for conventional pancreatic cancer remain unclear. Regarding the efficacy of adjuvant therapy for invasive IPMC, prior small‐scale retrospective studies have shown inconsistent results [7, 8, 9, 10, 11], leaving its effectiveness uncertain. Recently, findings from large‐scale retrospective studies have been reported [5, 12, 13, 14]. This review article introduces these studies.

Several RCTs have reported on the efficacy of neoadjuvant therapy (NAT) before surgery for conventional pancreatic cancer [15, 16, 17, 18, 19]. It has been demonstrated to be beneficial in prolonging survival for borderline resectable (BR) conventional pancreatic cancer with invasion of the portal vein/superior mesenteric vein (PV/SMV), celiac artery, or superior mesenteric artery (SMA) [15, 16]. On the other hand, for resectable conventional pancreatic cancer, the outcomes vary among studies [16, 17, 18, 19], and its utility remains a topic of debate. Regarding NAT for invasive IPMC, there are very few reports available. This review article introduces two recently published studies on this topic [5, 20].

Studies on postoperative recurrence of invasive IPMC following surgical resection have been increasing in recent years [2, 3, 21], revealing that recurrence occurs frequently even after curative resection. Recently, the results of large‐scale studies on recurrence patterns and treatments for invasive IPMC have been reported [4, 5], and these findings are introduced in this review article. This review aims to provide the latest information on multimodal treatment strategies for invasive IPMC and contribute to improving the long‐term survival of patients with invasive IPMC.

## 2 Surgical Resection

The standard treatment for invasive IPMC is surgical resection. However, even with surgical resection, postoperative recurrence may occur, and the prognosis is significantly worse compared to LGD and HGD [2, 22]. We encountered a case of invasive IPMC with an invasion length of only 700 μm that exhibited lymph node metastasis and developed lung metastasis despite completing adjuvant chemotherapy for 6 months after R0 radical surgery. According to reports from large‐scale retrospective studies, lymph node metastasis in invasive IPMC with an invasion length of less than 5 mm occurs in 3%–9% of cases, and the recurrence rate is 17%–19% [3, 23, 24]. Thus, our case is not uncommon. Therefore, it is crucial to consider surgical strategies for invasive IPMC. In surgery for invasive IPMC, the “pancreatic resection margin” and the “extent of lymph node dissection” are critical considerations. This chapter discusses these two key points based on recent literatures.

In surgery for invasive IPMC, the clinical questions remain: how should the pancreatic resection margin be determined, and is intraoperative frozen section pathological diagnosis necessary? A recent international multicenter retrospective study reported from the United States (US) analyzed 407 resected cases of invasive IPMC [25]. The pathological diagnosis of the pancreatic resection margin showed no dysplasia in 242 cases (59%), LGD in 123 cases (30%), HGD in 15 cases (4%), and invasive IPMC in 27 cases (7%) [25]. The study reported that cases with LGD at the resection margin did not show significant differences in overall survival or recurrence‐free survival compared to cases with no dysplasia [25]. However, cases with HGD or invasive IPMC at the resection margin of the pancreas had significantly worse survival outcomes [25]. Furthermore, among cases where HGD or invasive IPMC was detected by intraoperative frozen section diagnosis and additional resection was performed to achieve LGD or no dysplasia at the margin, survival outcomes were significantly better than those with HGD or invasive IPMC remaining at the margin [25].

Additionally, a study from Korea analyzed 548 resected IPMN cases (LGD: 353 cases, HGD: 78 cases, invasive IPMC: 117 cases) [26]. The pathological diagnosis of the pancreatic resection margin showed that cases with HGD or invasive IPMC had significantly higher 5‐year recurrence rates (50.6% vs. 8.3% vs. 5.9%, *p* < 0.001) and significantly worse 5‐year survival rates (30.8% vs. 80.7% vs. 83%, *p* < 0.001) compared to cases with no dysplasia or LGD [26]. The study concluded that additional resection is necessary until the resection margin achieves no dysplasia or LGD [26].

These findings support the recommendations in the 2024 International Consensus Guidelines for IPMN [1], which advocate for intraoperative frozen section pathological diagnosis of the pancreatic resection margin and additional resection if HGD or invasive IPMC is detected at the margin.

The next clinical question regarding invasive IPMC surgery concerns the optimal extent of lymph node dissection. For IPMNs with LGD or HGD, lymph node metastasis is theoretically not possible, and therefore, lymph node dissection is unnecessary, making less extensive surgery acceptable. However, in cases of invasive IPMC, even in T1a cases with an invasion length of less than 5 mm, lymph node metastasis can occur [3, 23, 24]. Cases with positive lymph node metastasis are associated with poor prognosis [2, 5, 21].

Thus, it is believed that sufficient lymph node dissection may be necessary in cases diagnosed for invasive IPMC preoperatively. This is reflected in the recommendation for “radical pancreatectomy with lymph node dissection” in the 2024 International Consensus Guidelines for IPMN [1]. However, given the rarity of invasive IPMC compared to conventional pancreatic cancer, robust evidence is lacking. Here, we introduce a recent large‐scale international multicenter retrospective study reported from US.

A correlation analysis between overall survival and the number of dissected lymph nodes in 341 cases of resected invasive IPMC identified the optimal number associated with improved overall survival as 20 [27]. The study reported that patients who had 20 or more lymph nodes dissected had significantly better survival compared to those with fewer than 20 lymph nodes dissected (80.3 months vs. 37.2 months, *p* < 0.001) [27]. These findings strongly suggest that lymph node dissection in invasive IPMC may improve survival, supporting the recommendation for radical pancreatectomy with lymph node dissection in the 2024 International Consensus Guidelines for IPMN [1].

However, the ability to accurately diagnose invasive IPMC preoperatively remains a critical challenge. For example, symptomatic LGD or HGD are indications for surgery, but lymph node dissection is unnecessary in these cases. If a preoperative diagnosis of LGD or HGD is made but the final pathological diagnosis reveals invasive IPMC, the lymph node dissection may be inadequate. Although many studies have identified predictive factors for HGD/invasive IPMC as indications for surgery [28, 29, 30], research on predictive factors specifically for invasive IPMC remains limited [30, 31, 32, 33]. This represents a significant area for future investigation.

## 3 Postoperative Adjuvant Therapy

Surgical resection is the only curative treatment for invasive IPMC; however, the recurrence rate after surgery remains high, and prognosis is poor once recurrence occurs. This raises the clinical question of whether postoperative adjuvant therapy could prolong survival in patients with invasive IPMC. Due to the significantly lower frequency of invasive IPMC compared to conventional pancreatic cancer, the effectiveness of postoperative adjuvant therapy for invasive IPMC had previously been examined in small‐scale retrospective studies [7, 8, 9, 10, 11] until two meta‐analysis papers were reported in 2022 [34, 35]. Subsequently, results from large‐scale, multicenter studies were reported from the United Kingdom (UK) [13], US [14], Korea [12], and Japan [5]. This chapter reviews the usefulness of postoperative adjuvant chemotherapy for invasive IPMC based on these four studies.

Four large‐scale, multi‐center, retrospective studies investigated the impact of postoperative adjuvant chemotherapy on survival in patients with invasive IPMC (Table 1). The studies included 332 cases from Korea, 459 cases from UK, 847 cases from US, and 1143 cases from Japan [5, 12, 13, 14]. All studies found no difference in survival between the groups that received adjuvant therapy after surgery and those that did not when examining all invasive IPMC cases [5, 12, 13, 14]. In both the Korean and UK studies, adjuvant chemotherapy was also not found to be beneficial for advanced cases of invasive IPMC [12, 13]. However, the US study reported that patients with poor prognostic factors, specifically lymph node metastasis and high preoperative serum CA19‐9 levels, experienced an extension in overall survival when adjuvant chemotherapy after surgery was administered (Table 1) [14].

**TABLE 1:** The usefulness of postoperative adjuvant chemotherapy for invasive IPMC based on recent large‐scale retrospective studies.

Author	Choi [12]	Hirono [5]	Lucocq [13]	Habib [14]
(Year)	(2023)	(2024)	(2024)	(2024)
Country	Korea	Japan	UK	USA
Time period reviewed	2001‐2020	1996‐2018	2010‐2020	2005‐2018
Number of patients with invasive IPMC	289	1,143	459	847
Adjuvant therapy (+)	157	486	275	538
Adjuvant therapy (−)	132	657	184	309
Benefit of adjuvant therapy for all patients	No	No	No	No
Patients having oncological benefits by adjuvant therapy	No	Stage IIB/III, high CA19‐9, lymphovascular invasion, perineural invasion, serosal invasion, or lymph nodes metastasis	No	Lymph nodes metastasis and high CA19‐9

Abbreviations: CA, carbohydrate antigen; IPMC, intraductal papillary mucinous carcinoma.

While these three studies analyzed survival based solely on the presence or absence of postoperative adjuvant therapy, the Japanese multi‐center study compared overall survival and recurrence‐free survival between patients who completed 6 months of adjuvant therapy and those who did not [5]. The Japanese study found that for patients with UICC stage IIB and III, high preoperative serum CA19‐9, lymphovascular invasion, perineural invasion, or lymph node metastasis, completing adjuvant therapy for 6 months led to improved overall survival and recurrence‐free survival [5]. Despite the overall lack of evidence supporting the effectiveness of postoperative adjuvant chemotherapy for all invasive IPMC cases, these large‐scale clinical studies suggest that adjuvant therapy after surgery may prolong survival in advanced cases.

Furthermore, studies from the UK and Japan also analyzed the chemotherapy regimens used. In the UK study, out of 275 patients who received adjuvant therapy, 141 (51%) were given gemcitabine (GEM), 60 (22%) received GEM and capecitabine combination therapy, 22 (8%) received FOLFIRINOX, and 52 (19%) received other regimens [13]. The study reported no differences in overall survival and recurrence‐free survival among the various regimens [13]. In the Japanese multi‐center study, of the 333 patients who completed adjuvant therapy after surgery, 226 (68%) received S‐1, 97 (29%) received GEM, and 10 (3%) received GEM and S‐1 combination therapy [5]. Patients treated with S‐1 had better overall survival and recurrence‐free survival compared to those treated with GEM or the GEM and S‐1 combination [5]. Although the JASPAC01 study demonstrated the efficacy of S‐1 as adjuvant therapy for conventional pancreatic cancer [36], similar effects might be present for invasive IPMC.

These large‐scale retrospective studies have reported the impact of postoperative adjuvant chemotherapy on survival in invasive IPMC. However, significant patient selection biases and differences in study results make the conclusions unclear. To identify the population that would benefit from adjuvant therapy after surgery and determine the optimal regimen and duration, multi‐center prospective RCTs are necessary.

## 4 Neoadjuvant Therapy (NAT)

Various RCTs have been conducted to evaluate the effectiveness of NAT for conventional pancreatic cancer, but its utility remains a subject of debate. Surgical resection alone is the standard treatment for invasive IPMC, yet reports on the effectiveness of NAT are extremely limited. Therefore, the 2024 International Consensus Guidelines for the management of IPMN do not include any recommendations regarding NAT for invasive IPMC [1]. This article introduces two recently published studies on NAT for invasive IPMC [5, 20].

In a multi‐institutional retrospective study reported from US, 25% of 105 cases of invasive IPMC underwent neoadjuvant therapy, compared to 65% of 1052 cases of conventional pancreatic cancer treated during the same period [20]. It was noted that the frequency of NAT was significantly lower for invasive IPMC than for conventional pancreatic cancer [20]. As this study is retrospective, the criteria for administering NAT remain unclear. According to the study results, the complete/partial response rates based on radiological RECIST criteria were 37% for conventional pancreatic cancer and 32% for invasive IPMC [20]. The stable disease rates were 51% for conventional pancreatic cancer and 62% for invasive IPMC, while the progressive disease rates were 7% and 4%, respectively, showing no significant differences between the two diseases [20]. Regarding pathological response, the marked response rates were 29% for conventional pancreatic cancer and 19% for invasive IPMC, with no significant difference observed between the two groups [20]. This study did not report the relationship between the use of NAT and survival outcomes in invasive IPMC. Therefore, the effectiveness of NAT for invasive IPMC remains unclear.

Another study on NAT for invasive IPMC is a multi‐institutional retrospective study from Japan. Among 1183 cases of invasive IPMC, only 40 cases (3%) underwent NAT [5]. After propensity score matching, overall and recurrence‐free survival after surgery were compared between 40 cases that received NAT and 40 cases that did not, revealing no significant differences in survival between the two groups [5]. However, in a subgroup analysis of 70 cases of BR invasive IPMC—defined as invasive components with abutment of less than 180° to the celiac artery or SMA, or abutment of 180° or more to the PV/SMV [37]—those who received NAT showed significantly better survival outcomes [5]. These findings suggest that NAT may be effective for BR invasive IPMC.

In previous RCTs evaluating the effectiveness of NAT for conventional pancreatic cancer, it has been reported that NAT significantly improves survival compared to surgery‐first approaches in BR conventional pancreatic cancer [15, 16]. However, for resectable conventional pancreatic cancer, the results of studies have varied [16, 17, 18, 19], and its utility remains a subject of ongoing debate. Regarding the effectiveness of NAT for invasive IPMC, no prospective clinical trials have been reported, and the current level of evidence remains extremely low. Furthermore, the optimal regimen and duration for NAT in invasive IPMC are entirely unknown, leaving many challenges to be addressed in the future.

## 5 Recurrence Pattern

There are several reports on recurrence patterns following surgical resection for invasive IPMC [2, 3, 4, 5, 21], although they are far fewer compared to those for conventional pancreatic cancer. Among them, two recent large‐scale multi‐institutional studies are particularly noteworthy.

The first report, from UK, analyzed 459 cases of invasive IPMC, identifying recurrence in 209 cases (46%) [4]. The recurrence patterns were reported as 30% liver metastases, 30% lung metastases, and 30% local recurrence [4]. Additionally, it was noted that postoperative adjuvant therapy and preoperative serum CA19‐9 levels were not associated with the occurrence of recurrence.

The second report, a multi‐institutional study from Japan, analyzed 1183 cases of invasive IPMC and found recurrence in 484 cases (41%) [5]. In this study, recurrences were classified into extra‐pancreatic recurrence and remnant pancreatic recurrence, observed in 33% and 8% of cases, respectively, with 5‐year cumulative incidence rates of 33% and 10% [5]. Among the 390 cases with extra‐pancreatic recurrence, the recurrence patterns were reported as follows: 27% had only local recurrence, 22% had only liver metastases, 18% had only lung metastases, 15% had only peritoneal dissemination, 1% had only bone metastases, and 17% had multiple recurrences [5]. On the other hand, among the 94 cases with remnant pancreatic recurrence, 67 developed IPMC, while 27 developed conventional pancreatic cancer.

In literatures comparing the recurrence patterns of invasive IPMC and conventional pancreatic cancer, a study reports that invasive IPMC is associated with a longer time to recurrence and overall survival from surgery, as well as a higher frequency of peritoneal dissemination and lung metastases but a lower frequency of local recurrence [38]. However, another study has found no significant differences in recurrence patterns between invasive IPMC and conventional pancreatic cancer [39], leaving the issue open to debate.

Nonetheless, the two large‐scale studies mentioned above reported that adjuvant therapy after surgery did not reduce recurrence frequency in all invasive IPMC cases [4, 5]. On the other hand, a study from Japan reported that in advanced invasive IPMC cases characterized by high preoperative serum CA19‐9 levels, positive lymphovascular invasion, positive perineural invasion, serosal invasion, and lymph node metastasis, postoperative adjuvant therapy improved recurrence‐free survival [5]. To clarify the postoperative recurrence patterns of invasive IPMC, further accumulation of cases and long‐term follow‐up are necessary.

## 6 Treatment for Recurrent Disease

The treatment of the recurrence diseases after surgical resection of invasive IPMC is not addressed in the 2024 International Consensus Guidelines for IPMN [1], and no established therapeutic approach currently exists. Here, we introduce two recent large‐scale studies analyzing postoperative recurrence of invasive IPMC.

The study reported from UK, included 209 cases (57%) of recurrence, among which 120 patients underwent some form of treatment [4]. The treatments consisted of chemotherapy in 92 cases, radiotherapy in 12 cases, surgical resection in 6 cases, and a combination of treatments in 10 cases [4]. Patients who received treatment for recurrence of invasive IPMC had significantly better overall survival (27 months vs. 14.6 months, *p* < 0.001) and survival from the time of recurrence diagnosis (12.3 months vs. 3.2 months, *p* < 0.001) compared to those who did not receive treatment [4].

Another study, a multicenter collaborative research conducted in Japan, analyzed 484 cases of recurrence after surgical resection for invasive IPMC [5]. Among these, 365 patients (75%) received treatment for recurrence [5]. The treatments included chemotherapy in 299 cases, surgical resection in 93 cases, and radiotherapy in 21 cases [5]. This study also reported that patients who received any treatment for recurrence had significantly better survival compared to those who did not (40.6 months vs. 22.4 months, *p* < 0.001) [5]. Furthermore, among 94 cases of remnant pancreatic recurrence, surgical resection significantly prolonged survival compared to those who did not undergo surgery (153.6 months vs. 69.9 months, *p* < 0.001) [5].

Based on the results of these large‐scale multicenter studies [4, 5], it is suggested that administering some form of treatment for recurrence after surgical resection for invasive IPMC may lead to prolonged survival. In other words, for invasive IPMC, severe postoperative follow‐up, similar to that for conventional pancreatic cancer, is essential. Prompt initiation of treatment upon detection of recurrence is expected to improve survival outcomes.

## 7 Conclusions

In the standard treatment of invasive IPMC, which is surgical resection, intraoperative pathological diagnosis of the pancreatic resection margin is useful. If the pancreatic resection margin shows HGD or invasive IPMC, additional pancreatic resection is necessary until the margin shows no dysplasia or LGD (Table 2). Regarding lymph node dissection, performing adequate lymph node dissection similar to that for conventional pancreatic cancer may help prevent recurrence and improve survival outcomes (Table 2). Adjuvant chemotherapy after surgery for invasive IPMC may be beneficial for advanced cases with lymph node metastasis, potentially preventing recurrence and prolonging survival (Table 2). Regarding NAT for invasive IPMC, there is little evidence; however, it may be useful in preventing recurrence and extending survival in cases of BR invasive IPMC with major vascular involvement (Table 2). For recurrence after surgical resection for invasive IPMC, timely treatment, similar to that for conventional pancreatic cancer, may improve survival outcomes (Table 2). This review introduced recent large‐scale studies on the treatment of invasive IPMC; however, as these are retrospective studies, the level of evidence remains low. Moving forward, conducting large‐scale prospective trials will be essential to establish effective multimodal treatments for invasive IPMC.

**TABLE 2:** A summary of recommendation about treatment for invasive IPMC based on recent large‐scale retrospective studies.

Factor	Suggestion based on recent studies
Surgical treatment	
Pancreatic resection margin	Additional resection is recommended until the margin of no dysplasia or low‐grade dysplasia if intraoperative frozen section pathological diagnosis of the pancreatic resection margin is high‐grade dysplasia or invasive IPMC
Extent of lymph node dissection	If invasive IPMC is suspected based on preoperative imaging or intraoperative diagnosis, regional lymph node dissection similar to that for conventional pancreatic cancer is recommended
Postoperative adjuvant therapy	For advanced invasive IPMC, completion of postoperative adjuvant therapy for 6 months may improve survival
Neoadjuvant therapy	Neoadjuvant therapy for invasive IPMC involving major vessels, including portal vein, celiac axis and/or superior mesenteric artery, may improve survival
Treatment for recurrent disease	Any treatment for recurrent disease after surgery for invasive IPMC is highly likely to improve survival
	In particular, surgical resection should be considered for remnant pancreatic recurrence

Abbreviations: CA, carbohydrate antigen; IPMC, intraductal papillary mucinous carcinoma.

## Author Contributions

**Seiko Hirono:** conceptualization, resources, supervision, validation, visualization, writing – original draft, writing – review and editing.

## Ethics Statement

The author has nothing to report.

## Consent

The author has nothing to report.

## Conflicts of Interest

The author S.H. is an Associate Editor of Annals of Gastroenterological Surgery.
